# Cover crop termination options and application of remote sensing for evaluating termination efficiency

**DOI:** 10.1371/journal.pone.0284529

**Published:** 2023-04-20

**Authors:** Vipin Kumar, Vijay Singh, Michael L. Flessner, Joseph Haymaker, Mark S. Reiter, Steven B. Mirsky

**Affiliations:** 1 Eastern Shore Agricultural Research and Extension Center, Virginia Tech, Painter, Virginia, United States of America; 2 School of Plant and Environmental Sciences, Virginia Tech, Blacksburg, Virginia, United States of America; 3 United States Department of Agriculture - Agricultural Research Service, Sustainable Agriculture Systems Laboratory, Beltsville, Maryland, United States of America; Indian Agricultural Research Institute, INDIA

## Abstract

Efficient termination of cover crops is an important component of cover crop management. Information on termination efficiency can help in devising management plans but estimating herbicide efficacy is a tedious task and potential remote sensing technologies and vegetative indices (VIs) have not been explored for this purpose. This study was designed to evaluate potential herbicide options for the termination of wheat (*Triticum aestivum* L.), cereal rye (*Secale cereale* L.), hairy vetch (*Vicia villosa* Roth.), and rapeseed (*Brassica napus* L.), and to correlate different VIs with visible termination efficiency. Nine herbicides and one roller-crimping treatment were applied to each cover crop. Among different herbicides used, glyphosate, glyphosate + glufosinate, paraquat, and paraquat + metribuzin provided more than 95% termination for both wheat and cereal rye 28 days after treatment (DAT). For hairy vetch, 2,4-D + glufosinate and glyphosate + glufosinate, resulted in 99 and 98% termination efficiency, respectively, followed by 2,4-D + glyphosate and paraquat with 92% termination efficiency 28 DAT. No herbicide provided more than 90% termination of rapeseed and highest control was provided by paraquat (86%), 2,4-D + glufosinate (85%), and 2,4-D + glyphosate (85%). Roller-crimping (without herbicide application) did not provide effective termination of any cover crop with 41, 61, 49, and 43% termination for wheat, cereal rye, hairy vetch, and rapeseed, respectively. Among the VIs, Green Leaf Index had the highest Pearson correlation coefficient for wheat (r = -0.786, p = <0.0001) and cereal rye (r = -0.804, p = <0.0001) with visible termination efficiency rating. Whereas for rapeseed, the Normalized Difference Vegetation Index (NDVI) had the highest correlation coefficient (r = -0.655, p = <0.0001). The study highlighted the need for tankmixing 2,4-D or glufosinate with glyphosate for termination instead of blanket application of glyphosate alone for all crops including rapeseed and other broadleaf cover crops.

## 1. Introduction

### 1.1 Cover crops and their termination

Cover crops have gained importance in sustainable cropping systems due to their potential benefits, such as reduced soil erosion and, enhanced nutrient cycling, water quality, and soil conservation [1–5]. The specific benefits cover crops provide depends on both the species (grasses, legumes and brassica) and the amount of biomass produced. Despite numerous benefits, growing cover crops, is associated with challenges, such as, ineffective termination at late growth stages, which may affect the emergence and yield of cash crops [6]. For example, approximately 12% yield reduction has been reported in corn (*Zea mays* L.) and soybean ((*Glycine max* L.) Merr.) due to incomplete termination of cereal rye (*Secale cereale* L.) and a cereal rye-legume cover crop mixture [7, 8]. Similarly, ineffective termination of hairy vetch, wheat (*Triticum aestivum* L.), annual ryegrass *(Lolium perenne* L. ssp *multiflorum* (Lam.) Husnot), and crimson clover (*Trifolium incarnatum* L.) can significantly reduce the soybean yield by 7–29% [9–11]. Poor termination of cover crops has also been reported to impact the growth and vigor of cash crops, especially early in the season, by secretion of allelochemical and depletion of soil moisture and nutrients [6].

In general, growers terminate cover crops utilizing three approaches: chemical, mechanical, and winter kill, depending on termination timing, cover crop species and crop growth stage. A nationwide survey of 1691 cover crop growers conducted in 2012 and 2013 indicated that 48% of growers use herbicides, 21% use mechanical methods, and 20% rely upon winter kill for termination of cover crops [12]. Mechanical termination methods include rolling, rolling-crimping, roll-chopping, undercutting, and mowing [13–15]. Roller-crimping is the most common mechanical tool used for assisting with cover crop termination. However, roller-crimpers are not effective for the termination of cover crops in the early season (March to mid-April) but are more effective as they reach maturity [16]. As such, employing roller-crimpers may lead to delayed planting of the cash crop. Winter kill of cover crops is also an option for cover crop termination of non-winter-hardy species, but is dependent on prevailing temperatures and is not a viable termination option for diverse locations and not effective in warmer winters [17]. Winter killing of cover crops also limits the benefits of cover crop planting, especially when grown for weed suppression benefits [18].

Owing to the listed issues associated with mechanical termination and winter kill, cover crop growers often prefer chemical termination strategies for effective termination of cover crops. In a survey conducted by Oliveira et al., [19] in Nebraska, it was observed that 95% of cover crop growers use herbicides for cover crop termination and similar trends have been observed in Delaware, Maryland and Virginia (DELMARVA) region (Mark VanGessel, personal communication). Chemical termination is achieved by various selective and non-selective herbicides before or after cash crop planting. The use of herbicides for cover crop termination also assists in controlling winter annual weeds growing alongside with cover crop plants. The termination efficiency of herbicides varies by cover crop species [20]. For instance, among various herbicides, glyphosate is found to be effective for termination of various grassy cover crop species such as cereal rye, winter wheat and annual ryegrass [6]. In a study conducted across five states (Arkansas, Indiana, Missouri, Mississippi and Wisconsin), Whalen et al., [9] reported 94–99% termination efficiency for cereal rye with the application of glyphosate alone or in combination with other herbicides. Similarly, in a study conducted in Arkansas, Palhano et al., [20] reported greater than 95% termination efficiency for cereal rye and wheat with the application of glyphosate. However, glyphosate is not effective for the termination of non-grass species [21, 22]. For instance, previous studies have found that termination efficiencies ranged from 56–69% in legumes and 58–86% for brassicas when using glyphosate [20]. Other popular herbicides such as 2,4-D and dicamba provide improved termination efficiencies for hairy vetch (80–87%) but very poor efficiencies for rapeseed (10–34%) [22]. However, glufosinate, another non-selective herbicide, showed the highest termination efficiency for hairy vetch (95%) [20], while showing a decreased efficiency (79%) compared to glyphosate (94–99%) for cereal rye [9]. Most of these studies evaluated non-selective herbicides like glyphosate, glufosinate and paraquat, which need to be used for cover crop termination before planting of the cash crops especially with non-herbicide resistant cash crops.

Recently, planting green (planting cash crops in standing cover crops) and then terminating cover crops one-to two-weeks after crop planting to allow higher cover crop biomass accumulation, weed suppression, and timely planting of cash crops, has grown in popularity. For instance, one study observed a 94–181% increase in cereal rye cover crop biomass with planting green as compared to planting brown (terminating cover crops before planting cash crop) [23]. Whereas, in another study, planting green led to 212–272 increase in cereal rye and wheat biomass and 12–28% increase in horseweed (*Conyza canadensis* L. Cronq.) control as compared with planting brown [24]. However, termination of cover crops in case of planting green scenario becomes an issue when non-glyphosate and glufosinate resistant crops are planted. Therefore, it is important to evaluate some selective herbicide options and compare them with non-selective herbicides for termination of commonly grown cover crops.

### 1.2 Vegetative Indices (VI)

The most commonly used methods for estimating cover crop termination efficiency with the use of herbicides are visual ratings, counting plant survival rates after application, and estimating biomass percentage changes. However, these methods are time-consuming and labor-intensive indicating a need to develop rapid and cost-effective methods to estimate cover crop termination efficiency. The use of unmanned aerial vehicles (UAVs) is increasing rapidly in agricultural research [25]. UAV-mounted remote sensing instruments provide a reliable and more objective estimate of phenotypic variation in plants as compared to the visual assessments [26]. Healthy and well-growing green plant tissues strongly absorb wavelengths in the red portion of visible light (~620–700 nm), while reflecting the majority of incoming solar irradiance in the near-infrared (NIR) wavelengths (~701–1399 nm) [27]. Plant tissues also differ in the absorbance and reflectance of wavelengths in other regions of the optical spectrum (350–2500 nm), and the mathematical combination of absorbance and reflectance of light energy in different bands can be used to calculate vegetation indices [28]. For instance, the NIR ratio (reflectance NIR/reflectance RED) was the first vegetative index (VI) to be used and found to have a strong correlation with leaf area index [29]. Numerous VIs has been developed to estimate crop biophysical and biochemical characteristics. For example, the normalized difference vegetation index (NDVI), and the normalized difference red-edge index (NDREI) accurately estimate biomass in cereal grass cover crop species (up to 1500–1900 kg ha ^-1^) [30, 31]. Additionally, the green normalized differential index (GNDVI) was significantly correlation with cover crop biomass (r = 0.58) and leaf chlorophyll content (r = 0.4) [32].

Along with crop nutrition and health assessment, in recent times, vegetative indices and remote sensing tools have been used to evaluate the herbicide injury on crops [33]. Zhang et al., [34] used hyperspectral images to calculate the Herbicide Damage Ratio Index (HDRI) and Herbicide Damage Normalized Index (HDNI) for estimating dicamba injury on dicamba sensitive soybean and found that the extent of dicamba injury on soybean can be estimated with hyperspectral images with over 90% accuracy. In another study, Oseland et al., [33] used different vegetative indices for evaluating 2,4-D and dicamba injury and yield reduction for soybean and found that NDREI is the more accurate index in estimating yield loss. Huang et al., [35] found that NDVI can efficiently estimate cotton (*Gossypium hirsutum* L.) yield loss following glyphosate exposure. However, information on the use of VIs for estimating the cover crop termination efficiencies of different herbicides is limited. Herbicides used for cover crop termination cause a reduction in leaf area and growth, and changes in plant coloration that can be assessed with VIs. Therefore, this study was designed with two objectives: (1) To evaluate the efficacy of different herbicides for the termination of commonly grown cover crops (wheat, cereal rye, hairy vetch, and rapeseed) and (2) to correlate the visually estimated termination efficiency data with different VIs (NDVI, GNDVI, NDRE, etc.) to find the most effective indices for estimating cover crop termination efficiency.

## 2. Material and methods

### 2.1 Cover crop planting, termination, and experimental design

**Fig 1:**
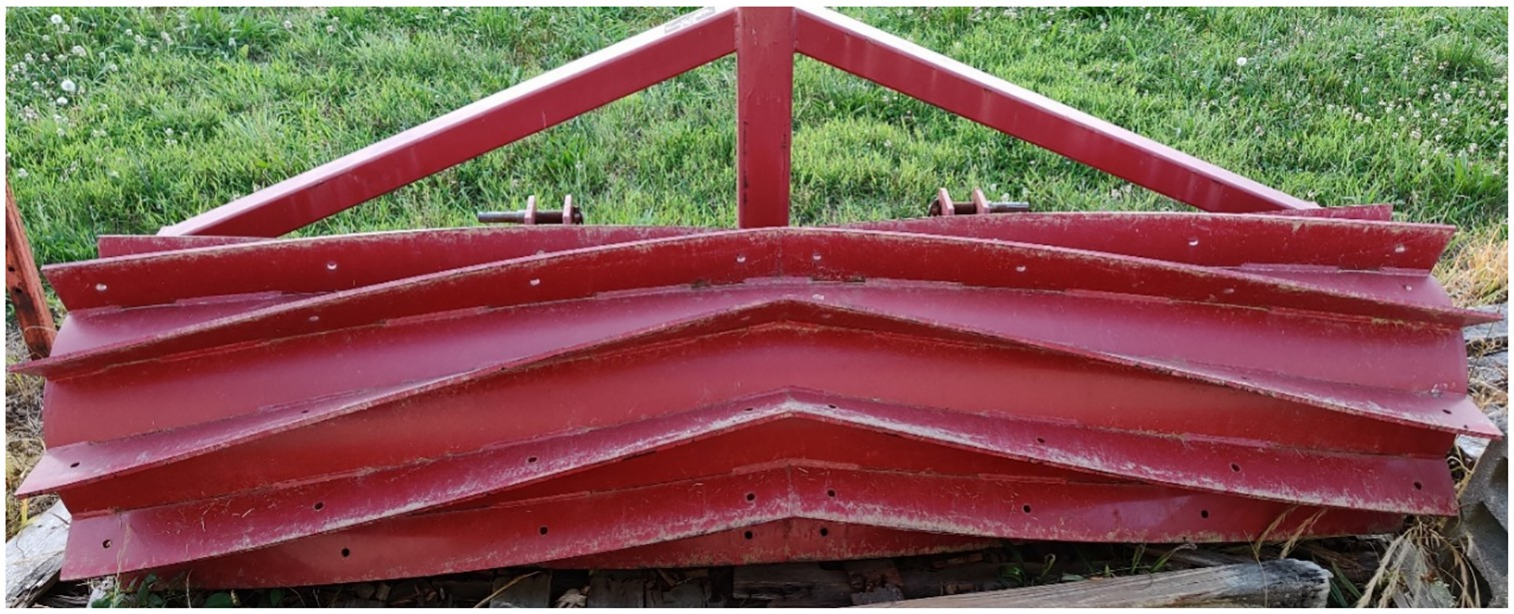
V-bar roller crimper (I&J Manufacturing, Gordonville, PA); 2.4 m in length, 38 cm diameter, 9.5 mm thickness, weighing 1002 kg used for rolling-crimping treatment.

**Fig 2:**
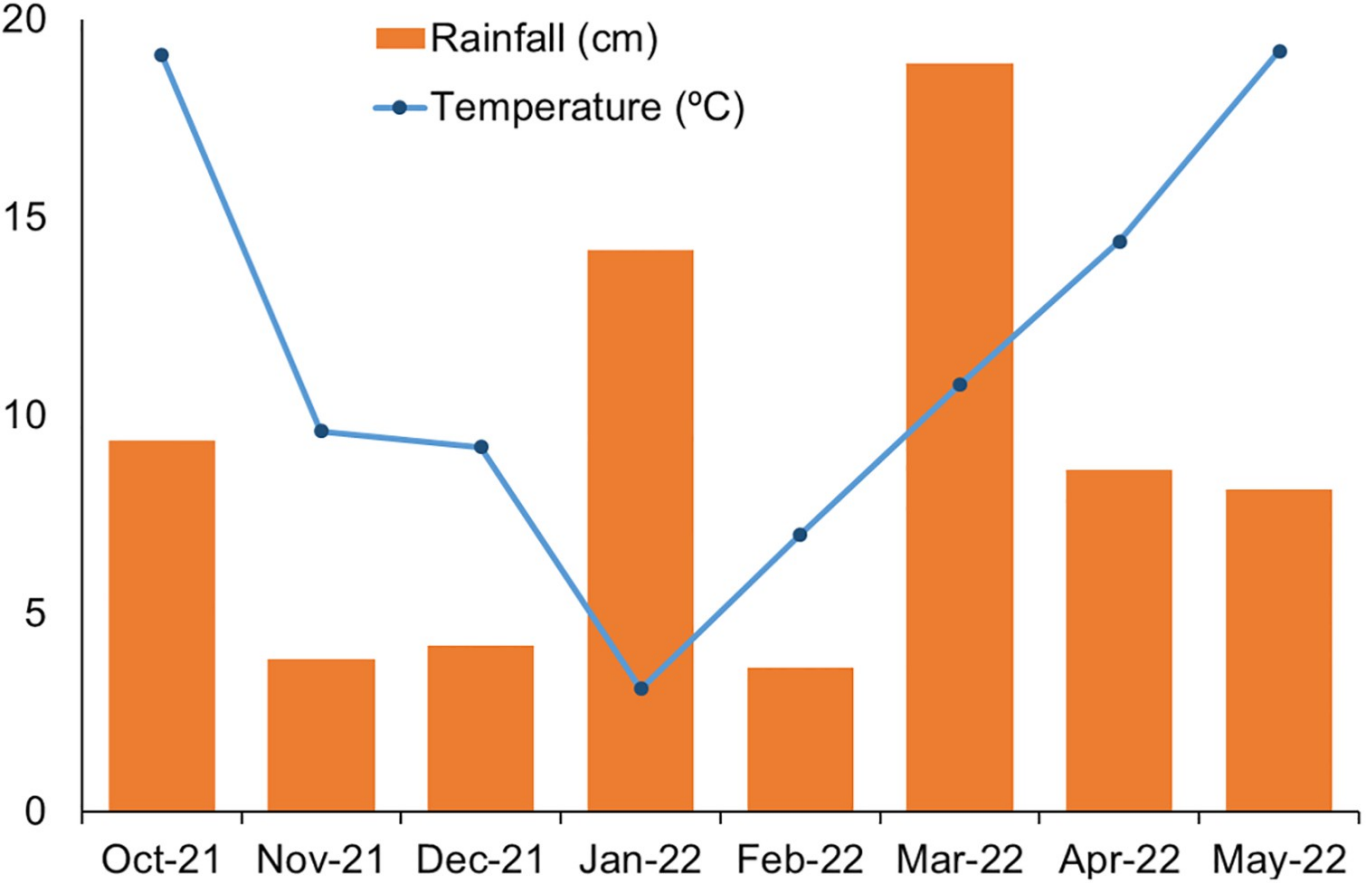
Monthly average temperature and rainfall during the experiment duration. Rainfall does not include 37cm snowfall received during Jan 2022.

The experiment was conducted at the Eastern Shore Agricultural Research and Extension Centre in, Painter, VA (37.5889, -75.8234) from October 2021-May 2022 on Bojac sandy loam soil (coarse-loamy, mixed, semiactive, thermic Typic Hapludults) with pH 5.7 and soil organic matter <1%. The study was repeated (two experimental runs) over time with a gap of 15 days in planting. The experiment was arranged in a randomized complete block design with four replications. Termination treatment included nine herbicide options and mechanical termination with roller-crimper (no-herbicide treatment). Termination treatment was a fixed factor and experimental run was kept as random factor. The plot size for each treatment was 2.43 m × 3.65 m. A V-bar roller-crimper (I&J Manufacturing, Gordonville, PA), 2.4 m in length, 38 cm diameter, 9.5 mm thickness, and weighing 1002 kg (Fig 1), was used in the experiment for rolling-crimping treatment. A check (no herbicide, only roller-crimper) treatment was included in the study for comparison purposes. Four cover crops species; wheat (*Triticum aestivum* L.), cereal rye (*Secale cereal* L.), hairy vetch (*Vicia villosa* Roth), and rapeseed (*Brassica napus* L.) were planted in strips of 2.43 m x 64 m on 5^th^ and 20^th^ October 2021, for the first and second experimental run, respectively. The field was tilled to prepare a fine seedbed before planting of cover crops. The respective cover crop seeding rate, variety, growth stage, and biomass at termination are listed in Table 1. Monthly rainfall and average temperature data throughout the experiment period are shown in Fig 2.

**Table 1:** Seeding rate, variety, and biomass of respective cover crop species at termination.

Cover crop species	Variety	Seed rate (kg/ha)	Biomass^a^ (t/ha)	
			Experimental run 1	Experimental run 2
Wheat	Dyna-Gro 9070	130	5.53	5.82
Cereal rye	Wrens Abruzzi	125	6.14	5.98
Hairy vetch	Aumerit	25	4.28	4.51
Rapeseed	Trophy Rape	8	3.46	3.37

^a^Biomass samples were collected from all the plots of respective cover crop species one day before termination, and represented as dry biomass after drying the samples for 6–7 days in a dryer at 65 ºC

For termination of cover crops, the herbicides were applied on April 10^th^ and April 25^th^, 2022, for the first and second run respectively, using a CO_2_- pressurized backpack sprayer. The sprayer was fitted with a 2.54 m wide boom, with 5 flat fan nozzles (XR8002) spaced 50.8 cm apart, delivering 187 L/ha of spray volume at 207 kPa. Suitable adjuvants were added as per the herbicide product label requirements. Herbicide product names and rates used for termination of respective cover crops have been given in Table 2.

**Table 2:** Herbicide product names, and rates used for termination of respective cover crop species. Adjuvants added with respective herbicides are shown in footnotes.

Cover crop species	Herbicide active ingredient	Herbicide product name	Rate (g ae or ai/ha)
Wheat and cereal rye	Clethodim^a^	Select Maxx^®^	275
	Glufosinate^b^	Liberty^®^	657
	Glyphosate^b^	Roundup Powermax^®^	1268
	Glufosinate + Glyphosate^b^	Liberty^®^ + Roundup Powermax^®^	1268 + 657
	Paraquat^c^	Gramaxone^®^	700
	Paraquat+metribuzin^c^	Gramaxone^®^ + Glory 4L^®^	560 + 262
	Quizalofop^c^	Provisia™	233
	Saflufenacil^d^	Sharpen^®^	100
	Sethoxydim^c^	Poast^®^	425
Rapeseed and hairy vetch	2,4-D^c^	LOW VOL 4	1068
	2,4-D + glufosinate^b^	LOW VOL 4+ Liberty^®^	534 + 657
	2,4-D + glyphosate^b^	LOW VOL 4 + Roundup Powermax^®^	534 + 1268
	Dicamba	XtendiMax^®^	560
	Glufosinate^b^	Liberty^®^	657
	Glyphosate^b^	Roundup Powermax^®^	1268
	Glyphosate+glufosinate^b^	Liberty^®^ + Roundup Powermax^®^	1268 + 657
	Paraquat^c^	Gramaxone^®^	700
	Thifensulfuron + tribenuron^c^	Harmony Extra^®^	24 + 12

^a^Non-ionic surfactant applied at 0.25% v/v

^b^Ammonium sulfate applied at 1% w/v

^c^Crop oil concentrate applied at 1% v/v

^d^Methylated seed oil applied at 1% v/v

Termination efficiency for all cover crop species was recorded 14 and 28 days after termination (DAT) on a 0–100 scale (0 = no termination and 100 = complete termination). Fresh and dry biomass of cover crop species was recorded one day before termination and 28 days after termination. Samples were kept at 65–70 °C for 6–7 days for the drying process. Percentage biomass reduction following herbicide application was calculated using the following Eq (1):

$$Percentage\;biomass\;reduction=\frac{\left(DWCk-DWTT\right)}{DWCk}\times 100$$ (1)

DWTT is the dry weight for respective termination treatment and DWCk is the dry weight of check treatment at 28 days after termination.

### 2.2 Aerial image acquisition

**Fig 3:**
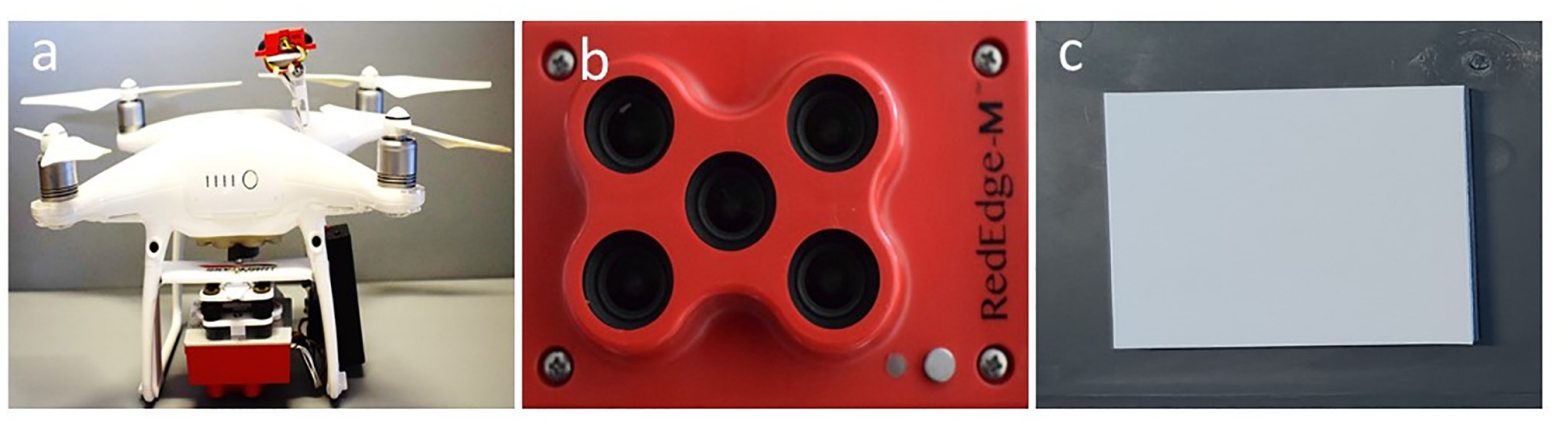
(a) Phatom-4 drone mounted with MicaSense RedEdge-M camera, (b) MicaSense RedEdge-M camera, and (c) Reflectance panel for radiometric calibration.

A Phantom 4 (Fig 3a) drone (DJI, Shenzhen, Guangdong, China) fitted with Micasense Rededge-M camera (MicaSense, Seattle, Washington, USA) was used to capture multispectral aerial images of cover crop plots, 28 DAT (Fig 3a). UAV flights were conducted at an altitude of 30 m above ground level with an operating speed of 2.3 m/s at noon (12–2 PM) time. The sky was clear, without any clouds when the images were taken. All images were collected vertically (0-3° from nadir), with 80% front and 70% side overlap. Micasense RedEdge-M camera (Fig 3b) captured images in five bands, blue (center 475 nm, bandwidth 20 nm), green (center 560 nm, bandwidth 20 nm), red (center 668 nm, bandwidth 10 nm), red-edge (center 717 nm, bandwidth 10 nm) and near-infrared (center 840 nm, bandwidth 40 nm). Pixel size was 0.8 cm and the shutter speed was below 2 ms^-1^ for all five bands. Drone flight parameters were kept constant for both experimental runs.

### 2.3 Aerial image processing and vegetation indices calculation

**Fig 4:**
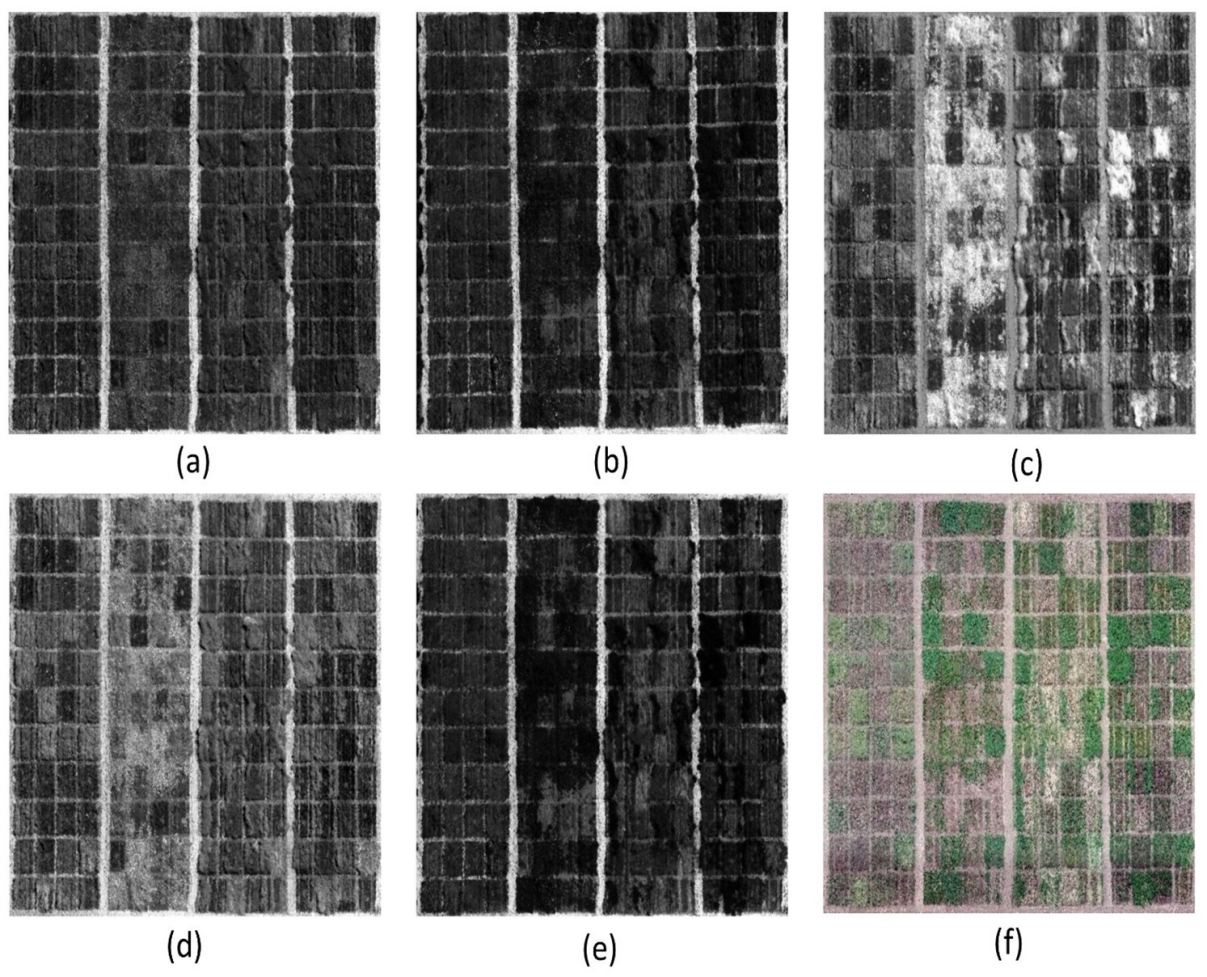
Reflectance map for respective bands and RGB image of the plots, (a) Blue, (b) Green, (c) NIR, (d) Red-edge, (e) Red, and (f) RGB image.

Drone flights produced 68 images for each experimental run, which were processed using Pix4DMapper (Pix4D SA, Lausanne, Switzerland) to generate a 16-bit orthomosaiced image. Radiometric calibration to reflectance was performed for solar irradiance and camera properties by using images of reflectance panel (Fig 3c) captured before the drone flight and respective irradiance values for different bands. Data images were processed using Pix4DMapper for automatic geometric correction. Pix4DMapper processed images in three steps: (1) identifying the overlapping points in the images and geo-rectifying images, (2) densifying the point cloud and generating mesh, and (3) generating digital surface model (DSM) and orthomosaicing images. Polygon with the ground size of 2.13 m × 3.35 m was generated for each experimental plot in collected imagery using QGIS 3.16 Hannover (QGIS Geographic Information System, Open-Source Geospatial Foundation Project). Each plot polygon was buffered by excluding 30 cm on all four sides to account for the edge effects. Reflectance values for all five bands (example, Fig 4) were then extracted from each plot polygon using the “Zonal Statistics as “Table tool” from toolbar and individual reflectance map generated by Pix4DMapper as “Input Value Raster”. Once reflectance value for each band was extracted from individual plot, formulas given in Table 3 were used to calculate respective VIs (Table 3).

**Table 3:** Vegetative indices calculated for estimating cover crop termination efficiency and their respective formula.

Vegetation Indices	Formula	Reference
Blue Normalized Difference Vegetation Index (BNDVI)	$\frac{rNIR-rBLUE}{rNIR+rBLUE}$	[36]
Blue Wide Dynamic Range Vegetation Index (BWDRVI)	$\frac{0.1\times rNIR-rBLUE}{0.1\times rNIR+rBLUE}$	[37]
Chlorophyll Index Green (CIG)	$\frac{rNIR}{rGREEN}-1$	[38]
Chlorophyll Index Red-edge (CIRE)	$\frac{rNIR}{rRedEdge}-1$	[38]
Green Blue Normalized Difference Vegetation Index (GBNDVI)	$\frac{rNIR-rGREEN+BLUE}{rNIR+rGREEN+BLUE}$	[36]
Green Leaf Index (GLI)	$\frac{2\times rGREEN-rRED-rBLUE}{2\times rGREEN+rRED+rBLUE}$	[39]
Green Normalized Difference Vegetation Index (GNDVI)	$\frac{rNIR-rGREEN}{rNIR+rGREEN}$	[40]
Green Ration Vegetation Index (GRVI)	$\frac{rNIR}{rGREEN}$	[41]
Normalized Green Red Difference Index (NGRDI)	$\frac{rGREEN-rRED}{rGREEN+rRED}$	[42]
Normalized Difference Red-edge Index (NDREI)	$\frac{rNIR-rRedEdge}{rNIR+rRedEdge}$	[40]
Normalized Difference Vegetation Index (NDVI)	$\frac{rNIR-rRED}{rNIR+rRED}$	[42]
Pan Normalized Difference Vegetation Index (PNDVI)	$\frac{rNIR-(rGREEN+rRED+rBLUE)}{rNIR+(rGREEN+rRED+rBLUE)}$	[36]

### 2.4 Statistics and data analysis

We conducted analysis of variance (ANOVA) on the visual estimates of termination efficiency 14 and 28 DAT and percentage biomass change 28 DAT for each species using SAS PROC MIX model (SAS Institute Inc., Cary, NC, USA). The effect of the experimental run and its interaction with termination treatment were found non-significant (Table 4), therefore, for final analysis, the experimental run was considered as a random effect. Means were separated using Fisher’s protected least significance difference (LSD) at 5% level of significance. Visible termination efficiency data 28 DAT was correlated with different vegetative indices (Table 3) values using SAS JMP Pro 16 (SAS Institute Inc., Cary, NC, USA), to evaluate vegetative indices that provide good estimate of termination efficiency of different herbicides. Two best performing indices were selected for each cover crops and linear regression was fitted for termination efficiency and respective indices value using SAS JMP Pro 16 to better understand the relationship between them.

**Table 4:** *P*-values for factors when experimental run was considered as a fixed effect.

		Wheat			Cereal rye			Hairy vetch			Rapeseed		
Source of variation	df	Visible termination^a^ (14 DAT^b^)	Visible termination (28 DAT)	Biomass reduction^a^	Visible termination (14 DAT)	Visible termination (28 DAT)	Biomass reduction	Visible termination (14 DAT)	Visible termination (28 DAT)	Biomass reduction	Visible termination (14 DAT)	Visible termination (28 DAT)	Biomass reduction
		p-values											
Expt run^c^	1	0.08	0.24	0.25	0.25	0.22	0.20	0.40	0.22	0.24	0.07	0.20	0.59
Termination treatment	9	<0.001	<0.001	<0.001	<0.001	<0.001	<0.001	<0.001	<0.001	<0.001	<0.001	<0.001	<0.001
Expt run × Termination treatment	9	0.25	0.47	0.82	0.08	0.19	0.06	0.14	0.22	0.23	0.66	0.79	0.66

^a^Visible termination (0–100 scale, 0 means no termination and 100 means complete termination) and biomass reduction was calculated in comparison to non-terminated check treatment. Non-terminated check treatment was excluded from data analysis

^b^DAT; Days after treatment

^c^Expt run = Experimental run

## 3. Results and discussion

### 3.1 Wheat

Paraquat provided effective termination (93%) of wheat 14 DAT (Table 5), and it was similar to paraquat + metribuzin (89%), glyphosate (88%), and glyphosate + glufosinate (88%). Termination efficiency with sole application of glufosinate (77%) was less than paraquat and paraquat + metribuzin. At 28 DAT, termination efficiency for paraquat, paraquat + metribuzin, glyphosate and glyphosate + glufosinate was greater than 95% (Table 5). Palhano et al., [20] also reported greater than 90% termination after 28 DAT with glyphosate and glyphosate + glufosinate. However, Palhano et al., [20] found less than 90% termination efficiency with paraquat and paraquat + metribuzin. Greater termination efficiency with paraquat and paraquat + metribuzin in this experiment as compared to Palhano et al., [20] could be attributed to greater spray volume used in this study (187 L/ha) as compared to 143 L/ha by Palhano et al., [20]. Paraquat being a contact herbicide, exhibits higher efficacy with increased spray coverage [43].

**Table 5:** Effect of termination treatment on termination efficiency and biomass change for wheat and cereal rye^a^.

		Wheat			Cereal rye		
Treatment	Rate (ai g ha^-1^)	Visible termination (14 DAT)	Visible termination (28 DAT)	Biomass reduction (%)	Visible termination (14 DAT)	Visible termination (28 DAT)	Biomass reduction (%)
Clethodim^b^	275	35d	40d	9de	34d	38c	7c
Glufosinate^c^	657	77b	78b	16c	93a	96a	27a
Glyphosate^c^	1268	88ab	97a	26ab	94a	97a	28a
Glufosinate + Glyphosate^c^	1268 + 657	88ab	95a	26ab	93a	96a	27a
Paraquat^d^	700	93a	96a	24b	99a	99a	26a
Paraquat+metribuzin^d^	560 + 262	89a	96a	29a	96a	98a	24a
Quizalofop^d^	233	48c	60c	16c	50bc	53bc	13bc
Saflufenacil^e^	100	36d	41d	11d	61b	64b	17b
Sethoxydim^d^	425	34d	42d	6e	41cd	43c	9c
Rolling-crimping^f^		34d	41d	10de	34d	61b	13bc

^a^Visible termination and biomass reduction means with a column followed by the same letter are statistically similar to each other at 5% level of significance

^b^Non-ionic surfactant applied at 0.25% v/v

^c^Ammonium sulfate applied at 1% w/v

^d^ Crop oil concentrate applied at 1% v/v

^e^Methylated seed oil applied at 1% v/v

^f^V-bar roller crimper (I&J Manufacturing, Gordonville, PA) was hollow from inside, 2.4 m in length, 38 cm diameter, 9.5 mm thickness, fully filled with water weighing 1002 kg used for rolling-crimping treatment

Among ACCase inhibitor herbicides used in the study, quizalofop provided greater termination, 48 and 60% at 14 and 28 DAT, respectively, as compared to clethodim (35 and 40%) and sethoxydim (34 and 42%). Childers et al., [44] reported more than 95% wheat injury with the application of quizalofop at early crop growth stages, however, in this study, 60% termination efficiency was observed. Lower termination efficiency with quizalofop application in this study can be attributed to the wheat growth stage as herbicides were applied for termination at booting stage. Other reason for lower termination efficiency with quizalofop could be prevailing high temperature conditions in this study as compared to study conducted by Childers et al., [44]. Metabolism of quizalofop increases at high temperatures resulting in lower wheat injury [45]. Reduced activity of other ACCase inhibiting herbicide activity at higher temperatures has been reported by Smeda and Putnam, [46] and Matzrafi et al., [47].

The effect of different termination treatments on biomass 28 DAT was also significant. Herbicides with greater than or equal to 95% termination efficiency (paraquat, paraquat + metribuzin, glyphosate, glyphosate + glufosinate) resulted in 24–29% biomass reduction (Table 5). Reduction in biomass with glufosinate and quizalofop was 16%, followed by saflufenacil (11%), whereas clethodim and sethoxydim caused less than 10% biomass reduction (Table 5). Use of the roller-crimper provided 41% termination efficiency and 10% biomass reduction 28 DAT as compared to >80% termination efficiency reported by Ashford and Reeves, [16]. Lower termination efficiency with the roller-crimper could be attributed to the use of roller-crimper at booting stage compared to anthesis and soft dough stage by Ashford and Reeves, [16]. Termination efficiency of wheat with roller-crimper significantly varies with wheat growth stage, with better termination at late growth stages [16].

### 3.2 Cereal rye

Termination efficiency for cereal rye was greater than 90% 14 DAT for paraquat, paraquat + metribuzin, glyphosate, glufosinate, and glyphosate + glufosinate. At 28 DAT, all of these herbicide treatments provided greater than 95% termination efficiency (Table 5). Cornelius and Bradley, [6] and Palhano et al., [20] also reported more than 90% termination efficiency with the use of glyphosate alone or in combination with glufosinate. However, both studies found less than 90% termination with glufosinate, and paraquat alone or in combination with a photosystem II (PS II)-inhibitor herbicides (atrazine and metribuzin). Greater termination efficiency in this study can be attributed to higher spray volume, which provided increased spray distribution and greater canopy distribution [43]. Saflufenacil provided 61 and 64% termination efficiency 14 and 28 DAT, respectively. Whereas termination efficiency with ACCase inhibitor herbicides; quizalofop, sethoxydim, and clethodim was 53, 43, and 38%, respectively, 28 DAT. Each of the three ACCase-inhibitors were statistically similar in terms of their efficacy (Table 5).

Termination treatments resulting in greater than 95% termination efficiency (paraquat, paraquat + metribuzin, glyphosate, glufosinate, and glyphosate + glufosinate) 28 DAT reduced the cereal rye biomass by 24–28%, but all of these treatments were statistically similar with each other (Table 5). Saflufenacil resulted in 17% reduction in biomass, followed by quizalofop (13%). Biomass reduction in sethoxydim and clethodim treated plots was less than 10% (Table 5). The biomass reduction results followed a trend similar to termination efficiency discussed above.

Termination efficiency with the roller-crimper remained at 34% 14 DAT, and increased to 61% 28 DAT (Table 5), which is lower than the recommended termination required (90% or greater) for sowing of cash crops into the cereal rye residues [48]. Greater than 90% termination efficiency of cereal rye at late growth stage with roller-crimping has been reported by Kornecki [49] and Mirsky et al., [50]. Lesser termination efficiency of the roller-crimper in our experiment can be attributed to use of roller-crimper at jointing and jointing-booting stage for experimental run one and two, respectively, as compared to Mirsky et al., [50] where cereal rye was terminated at soft dough stage. Cereal rye termination efficiency using a roller-crimper is dependent on the growth stage and terminating at late crop growth stage improves the termination efficiency [50]. Kornecki et al., [48] also reported lesser termination efficiency for cereal rye at early growth stage termination with a roller-crimper.

### 3.3 Hairy vetch

Termination efficiency of hairy vetch 28 DAT was greatest with 2,4-D + glufosinate (91%), which was statistically similar to glyphosate + glufosinate (89%), and paraquat (87%). Glufosinate, 2,4-D, 2,4-D + glyphosate, and dicamba provided 72–79% termination efficiency 14 DAT (Table 6). Whereas termination efficiency 14 DAT with glyphosate and thifensulfuron + tribenuron remained lowest (63%) among all herbicides used in the experiment. Roller-crimping resulted in 32% termination efficiency 14 DAT (Table 6). At 28 DAT, 2,4-D + glufosinate resulted in 99% termination efficiency, which was greatest among all termination treatments used for hairy vetch, but statistically similar to glyphosate + glufosinate, paraquat, and 2,4-D + glyphosate with 98, 92, and 92% termination efficiency, respectively (Table 6). Whalen et al., [9] also reported more than 90% termination efficiency for hairy vetch 28 DAT with 2,4-D + glufosinate and 2,4-D + glyphosate. The termination efficiency with paraquat was approximately 80% (12). Sole use of 2,4-D, dicamba and glufosinate provided 86, 87, and 84% termination efficiency 28 DAT, however the three herbicides were statistically similar (Table 6). At 28 DAT, thifensulfuron + tribenuron and glyphosate provided 79 and 74% termination efficiency (Table 6). Similar results were observed by Pittman et al., [22], where 80, 87, 81, and 69% termination efficiency at 28 DAT with 2,4-D, dicamba, glufosinate, and glyphosate was reported. All termination treatments reduced hairy vetch biomass 28 DAT. Maximum biomass reduction was observed in 2,4-D + glufosinate (44%) treated plots followed by glyphosate + glufosinate (43%). 2,4-D, dicamba, paraquat, and 2,4-D + glyphosate treated plots resulted in 32, 34, 38, and 39% biomass reduction 28 DAT, respectively. Biomass reduction 28 DAT was the greatest with hairy vetch among all cover crop species, which is attributed to rapid decomposition of dead plant tissue. Hairy vetch, a leguminous crop, has a low C:N ratio as compared to non-leguminous plants, and has a faster decomposition rate compared to non-leguminous cover crop species [51]. Faster decomposition rate for hairy vetch plant residues has been also reported by Sievers and Cook, [52]. Roller-crimping did not provide effective termination of hairy vetch 14 and 28 DAT, with only 32 and 49% termination efficiency, respectively (Table 6). Roller-crimping resulted in the lowest biomass reduction (11%). Greater termination efficiency for hairy vetch with roller-crimpers can be achieved if the crop is terminated at late flowering to early pod stage, whereas termination at vegetative-early flowering stage increases the chance for poor termination [53].

**Table 6:** Effect of termination treatment on termination efficiency and biomass change for hairy vetch and rapeseed^a^.

		Hairy vetch			Rapeseed		
Treatment	Rate (ai g/ha)	Visible termination (14 DAT)	Visible termination (28 DAT)	Biomass change (%)	Visible termination (14 DAT)	Visible termination (28 DAT)	Biomass change (%)
2,4-D^b^	1068	72c	86bc	32c	52ef	50e	7d
2,4-D + glufosinate^c^	534 + 657	91a	99a	44a	82a	85a	22a
2,4-D + glyphosate^c^	534 + 1268	78b	92ab	39ab	69b	85a	23a
Dicamba	560	76bc	87bc	34bc	36h	40f	6d
Glufosinate^c^	657	79b	84cd	26de	55de	60d	11bc
Glyphosate^c^	1268	63d	74e	23e	61cd	69c	13b
Glyphosate+glufosinate^c^	1268 + 657	89a	98a	43a	68bc	74bc	12bc
Paraquat^b^	700	87a	92ab	38ab	84a	86a	25a
Thifensulfuron + tribenuron^b^	24 + 12	63d	79de	30cd	43g	51e	8cd
Rolling-crimping^d^		32e	49f	11f	45fg	43f	9cd

^a^Visible termination and biomass reduction means with a column followed by the same letter are statistically similar to each other at 5% level of significance

^b^Crop oil concentrate applied at 1% v/v

^c^Ammonium sulfate applied at 1% w/v

^e^V-bar roller crimper (I&J Manufacturing, Gordonville, PA) which was hollow from inside, 2.4 m in length, 38 cm diameter, 9.5 mm thickness, fully filled with water weighing 1002 kg used for rolling-crimping treatment

### 3.4 Rapeseed

Paraquat and 2,4-D + glufosinate provided greatest termination efficiency, 84 and 82%, respectively, for rapeseed 14 DAT (Table 6). 2,4-D + glyphosate and glyphosate + glufosinate resulted into 69 and 61% termination efficiency, followed by a sole application of glyphosate (61%). Dicamba and 2,4-D (sole application) provided only 36 and 52% termination of rapeseed 14 DAT (Table 6). At 28 DAT, paraquat resulted in 86% termination efficiency followed by 2,4-D + glufosinate (85%) and 2,4-D + glyphosate (85%) termination efficiency (Table 6). Sole application of glyphosate and glufosinate provided 69 and 60% termination efficiency. However, 2,4-D + glyphosate and glyphosate + glufosinate resulted into 75 and 74% termination. Whereas 2,4-D and dicamba provided only 50 and 40% termination efficiency, respectively, 28 DAT (Table 6). Thifensulfuron + tribenuron provided 43 and 51% termination 14 and 28 DAT. Askew et al., [21] and Pittman et al., [22] reported 34–70%, 9–40%, 50–67%, and 50–68% termination efficiency with 2,4-D, dicamba, glufosinate, and paraquat, respectively. Greater termination efficiency with paraquat in this study is attributed to greater spray volume used. Askew et al., [21] reported similar termination efficiency with glyphosate + glufosinate (72%) to this study (74%) 28 DAT, however Askew et al., [21] reported greater termination efficiency with 2,4-D + glyphosate (96%) as compared to 85% in this study. As compared to the nontreated check treatment application of paraquat, 2,4-D + glyphosate, and 2,4-D+ glufosinate resulted in 22–25% biomass reduction 28 DAT (Table 6). Glyphosate, glufosinate, and glyphosate + glufosinate plots showed a biomass reduction of 11–13%, whereas thifensulfuron + tribenuron, 2,4-D, and dicamba caused less than 10% biomass reduction (Table 6). Termination efficiency and biomass reduction with roller-crimping was 43 and 9% 28 DAT, respectively (Table 6). Poor termination of rapeseed with roller-crimper is also reported by Price et al., [54].

### 3.5 Correlation of visible termination efficiency and vegetation indices

**Fig 5:**
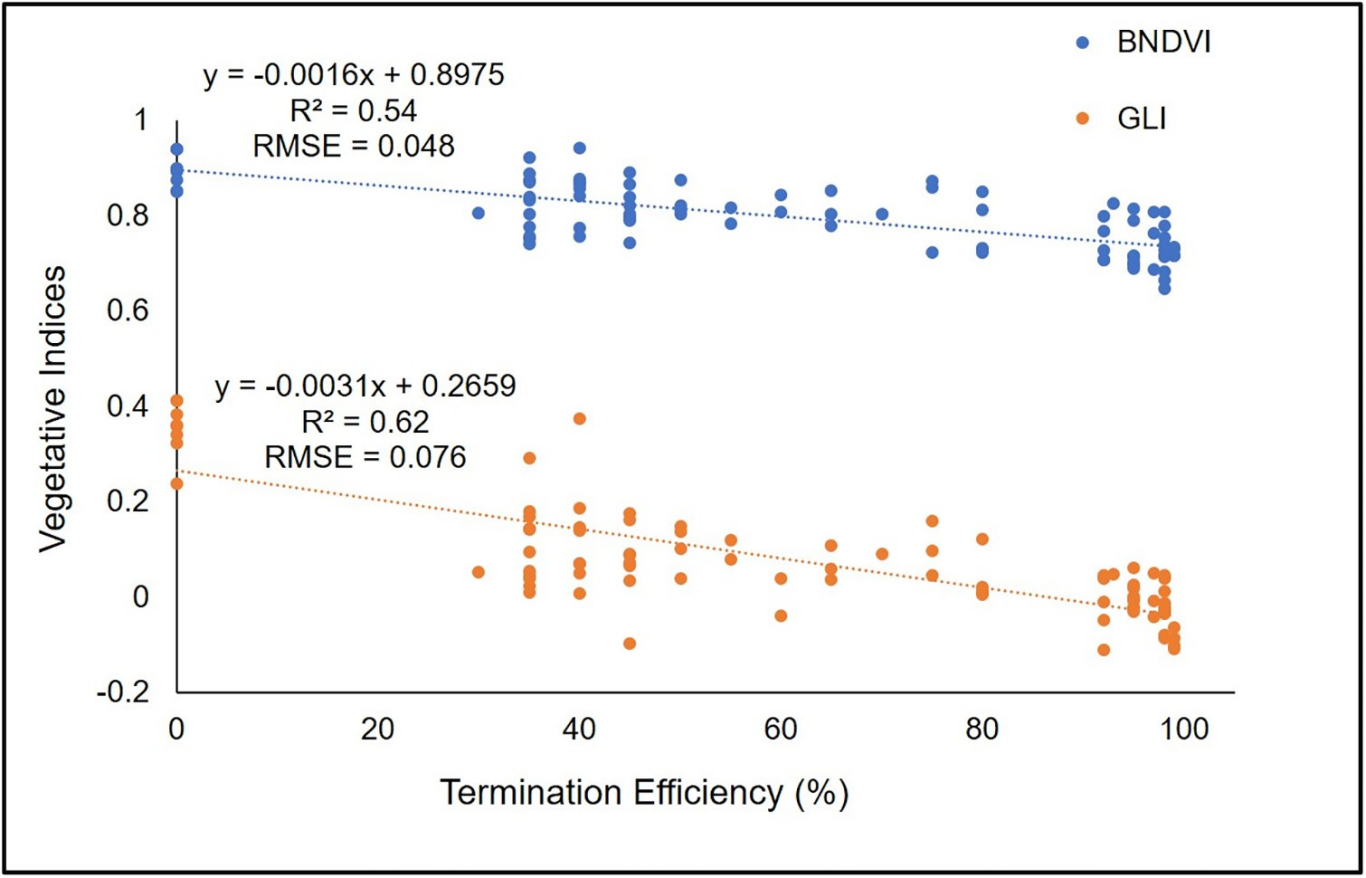
Scatter plot of Blue Normalized Difference Vegetation Index (BNDVI) and Green Leaf Index (GLI) response to termination efficiency (n = 88 for each vegetative indices) in wheat. R^2^ = Coefficient of determination, RMSE = Root Mean Square Error.

**Fig 6:**
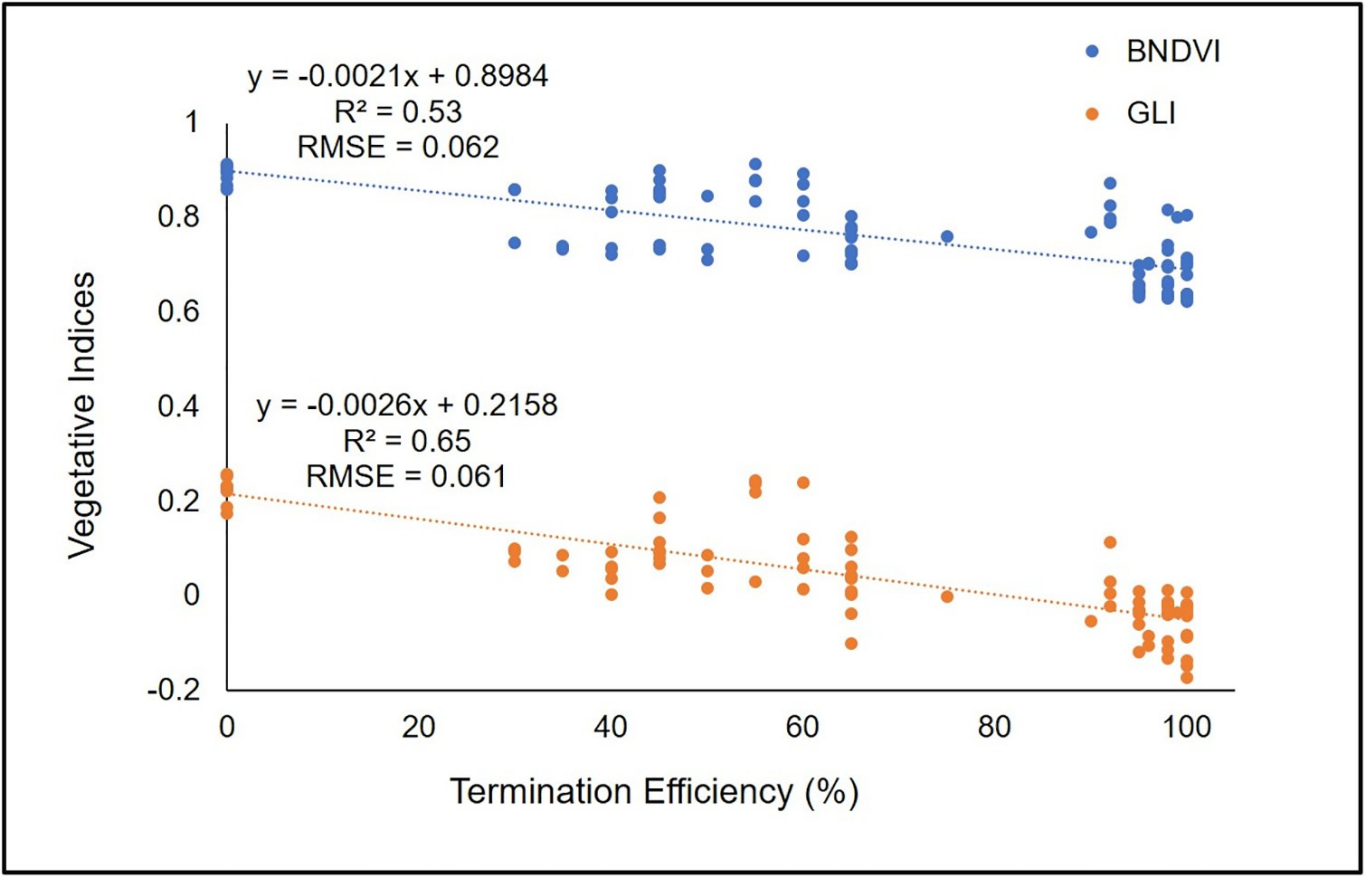
Scatter plot of Blue Normalized Difference Vegetation Index (BNDVI) and Green Leaf Index (GLI) response to termination efficiency (n = 88 for each vegetative indices) in cereal rye. R^2^ = Coefficient of determination, RMSE = Root Mean Square Error.

For all species, the vast majority of VIs decreased as the termination efficiency increased. For wheat and cereal rye, Pearson correlation coefficients between VIs and visible termination efficiency rating ranged from -0.51 to -0.79 and -0.52 to -0.80, respectively, and were all statistically significant (p <0.05) (Table 7). Among different VI, Green Leaf Index (GLI) and Blue Normalized Difference Vegetation Index (BNDVI) had the highest value for correlation coefficient, -0.79 (p <0.0001) and -0.73 (p <0.0001) for wheat and -0.80 (p <0.0001) and -0.72 (p <0.0001) for cereal rye, respectively. Therefore, linear regression equation was fitted for GLI and BNDVI to further quantify the VI values and visible termination efficiency rating in wheat (Fig 5) and cereal rye (Fig 6). Linear regression of GLI with wheat visible termination efficiency resulted in coefficient of determination (R^2^) 0.62 and an RMSE of 0.076. (Fig 5). Similarly, for cereal rye the value of R^2^ and RMSE was 0.65 and 0.061, respectively for GLI (Fig 6). This indicates that GLI is a better VI for estimation of termination efficiency as compared to BNDVI. This has been corroborated by Hunt Jr. et al., [39], where they found better correlation of GLI as compared to other VI with total leaf chlorophyll content.

**Table 7:** Value of Pearson correlation coefficient between termination efficiency and vegetative indices for respective cover crop species.

Vegetative indices	Wheat	Cereal rye	Hairy Vetch	Rapeseed
BNDVI	-0.73	-0.72	-0.11^ns^	-0.30
BWDRVI	-0.70	-0.68	-0.13^ns^	-0.31
CIG	-0.51	-0.57	-0.19^ns^	-0.44
CIRE	-0.62	-0.58	-0.17^ns^	-0.38
GBNDVI	-0.64	-0.61	-0.20^ns^	-0.56
GLI	-0.79	-0.80	0.02^ns^	-0.12^ns^
GNDVI	-0.61	-0.52	-0.20^ns^	-0.42
GRVI	-0.58	-0.57	-0.18^ns^	-0.45
NGRDI	-0.68	-0.70	0.01^ns^	-0.30
NDREI	-0.62	-0.56	-0.19^ns^	-0.36
NDVI	-0.62	-0.67	-0.11^ns^	-0.66
PNDVI	-0.67	-0.65	-0.16^ns^	-0.62

^ns^Non-significant at 5% level of significance, correlation coefficient values not followed by ns are significant at 5% level of significance

BNDVI = Blue Normalized Difference Vegetation Index, BWDRVI = Blue Wide Dynamic Range Vegetation Index, CIG = Chlorophyll Index Green, CIRE = Chlorophyll Index Red-edge, GBNDVI = Green Blue Normalized Difference Vegetation Index, GLI = Green Leaf Index, GNDVI = Green Normalized Difference Vegetation Index, GRVI = Green Ration Vegetation Index, NGRDI = Normalized Green Red Difference Index, NDREI = Normalized Difference Red-edge Index, NDVI = Normalized Difference Vegetation Index, PNDVI = Pan Normalized Difference Vegetation Index.

**Fig 7:**
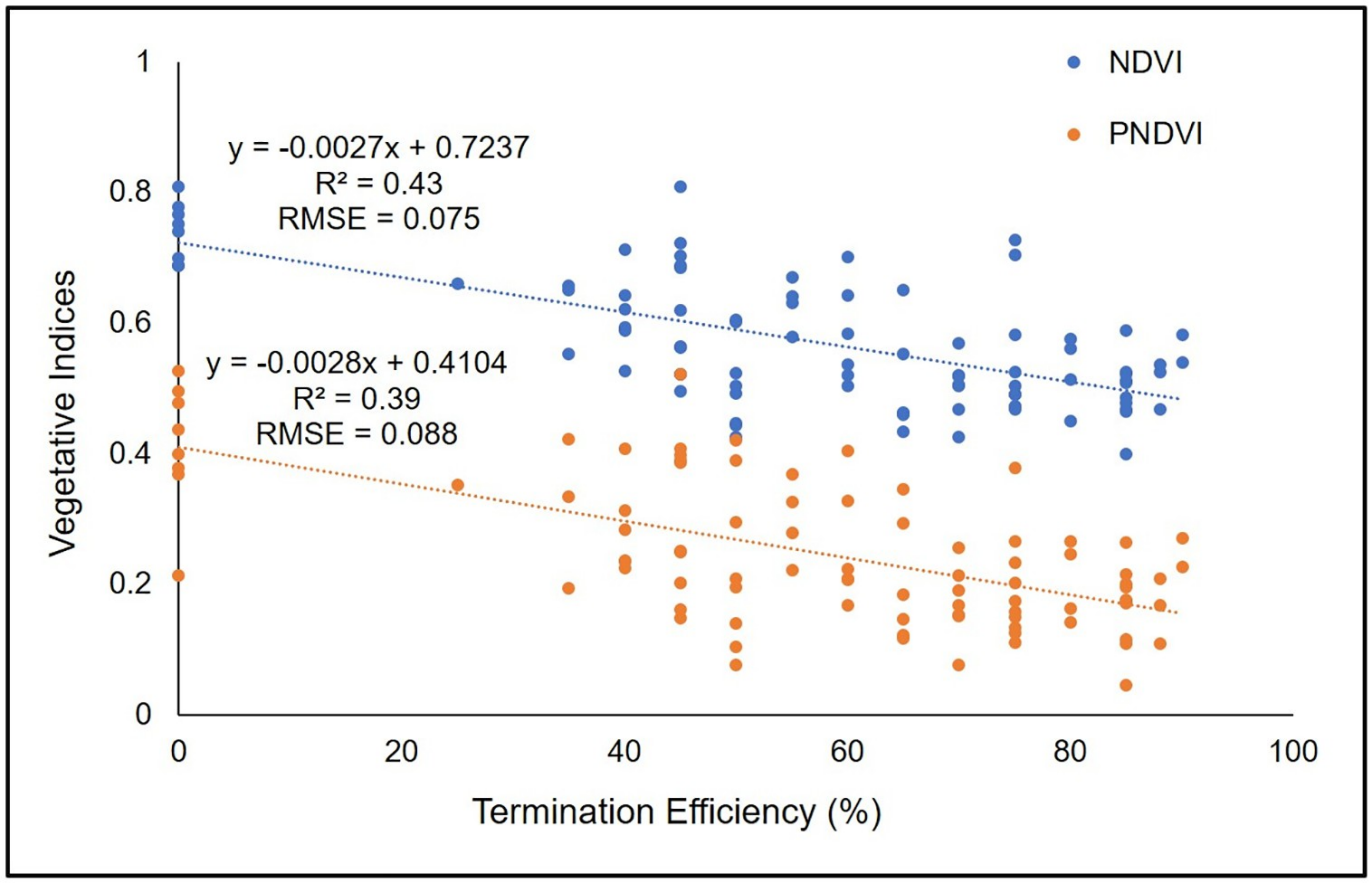
Scatter plot of Normalized Difference Vegetation Index (NDVI) and Pan Normalized Difference Vegetation Index (PNDVI) response to termination efficiency (n = 88 for each vegetative indices) in rapeseed. R^2^ = Coefficient of determination, RMSE = Root Mean Square Error.

In contrast to wheat and cereal rye, all VIs except GLI had a significant correlation coefficient for visible termination efficiency for rapeseed (Table 7). NDVI (r = -0.66, p<0.0001) had the highest correlation coefficient value for rapeseed visible termination efficiency ratings, followed by PNDVI (r = -0.62, p<0.0001). Linear regression equation was fit for NDVI and PNDVI, for rapeseed visible termination efficiency (Fig 7). Linear regression equation for visible rapeseed termination efficiency resulted in R^2^ of 0.43 and 0.39 and RMSE of 0.075 and 0.088 for NDVI and PNDVI, respectively. However, none of the VIs had a correlation coefficient greater than -0.7 for termination efficiency of rapeseed. This is likely due to the fact that some rapeseed plots included plants with yellow flowers which influenced the reflectance and VI values. Shen et al., [55] also reported the effect of yellow flowers from *Halerpestes tricuspis* on NDVI and Enhanced Vegetation Index (EVI). Yellow colored flowers increase reflectance in green-red wavelength, but do not affect blue and near infra-red wavelengths, thereby influencing VI values [55]. Similarly, Dixon et al., [56] found that VIs based on plant greenness decreases with appearance of flowering in plants. In hairy vetch, no VIs were significantly correlated with visible termination rating, which is likely due to an infestation of curly dock (*Rumex crispus* L.) in a number of hairy vetch plots which had greater termination efficiency. This infestation caused the well terminated plots to appear green and therefore influenced the surface reflectance.

To date, VIs have not been used for evaluating the cover crop termination efficiency with herbicides, but overall blue reflectance-based VIs were found to be better than normal green reflectance-based vegetation indices for estimation of termination efficiency in our study (Table 7) for grass cover crop species. Blue band-based VIs have been reported better for estimation of leaf area, biomass, and yield specially at late crop growth stages because of saturation of green reflectance based VI [36, 37, 57]. However, additional studies are required to evaluate effectiveness of GLI and other blue reflectance-based VIs for estimating the termination of other grass cover crops species at different termination timings.

## 4. Conclusion

This study indicates that efficacy of selective herbicides for termination of wheat, cereal rye, hairy vetch, and rapeseed was significantly lower than non-selective herbicides alone or combination of selective and non-selective herbicides. For rapeseed, none of the herbicide treatments provided more than 90% termination efficiency. This necessitates additional studies with other herbicides alone or in combination with roller-crimper for effective termination of rapeseed. We also found that VIs (GLI and BNDVI) were useful for estimating the termination efficiency of grass cover crop species but further studies are required to understand why GLI and BNDVI performed better than other vegetation indices in estimating cover crop termination efficiency. Whereas for rapeseed, NDVI and PNDVI were found more efficient than other vegetation indices for estimating termination efficiency, but still had poor relationships overall. Results from this study indicate that vegetative indices can provide estimates of the cover crop termination efficiency in cereal cover crops and have the potential to be further applied for weed control evaluations later in the season.

## Supporting information

S1 File Data indicating impact of herbicides on cover crop biomass and corresponding vegetative indices. (XLSX)

## References

[1] Reicosky DC, Forcella F. Cover crop and soi lquality interactions in agroecosystems. Journal of Soil and Water Conservation. 1998 JULY;53(3):6.

[2] Teasdale J, Brandsaeter L, Calegari A, Neto F. Cover crops and weed management. Upadhyaya M, Blackshaw R, editors. Wallingford, UK: CABI; 2007 Oct 1.

[3] Edward L, Burney J. Cover crops. Daniel Hillel JLH, editor: Elesvier Science; 2005.

[4] Cornelius CD, Bradley KW. Influence of Various Cover Crop Species on Winter and Summer Annual Weed Emergence in Soybean. Weed Technology. 2017;31(4):503–13. doi: 10.1017/wet.2017.23

[5] Yeo IY, Lee S, Sadeghi AM, Beeson PC, Hively WD, McCarty GW, et al. Assessing winter cover crop nutrient uptake efficiency using a water quality simulation model. Hydrology and Earth System Sciences. 2014;18(12):5239–53. doi: 10.5194/hess-18-5239-2014

[6] Cornelius CD, Bradley KW. Herbicide Programs for the Termination of Various Cover Crop Species. Weed Technology. 2017;31(4):514–22. doi: 10.1017/wet.2017.20

[7] Eckert D. Rye cover crops for no-tillage corn and soybean production. Journal of Production Agriculture. 1988;1(3):272–4.

[8] Mitchell WH, Teel MR. Winter-Annual Cover Crops for No-Tillage Corn Production¹. Agronomy Journal. 1977;69(4):569–73. doi: 10.2134/agronj1977.00021962006900040011x

[9] Whalen DM, Bish MD, Young BG, Conley SP, Reynolds DB, Norsworthy JK, et al. Herbicide programs for the termination of grass and broadleaf cover crop species. Weed Technology. 2019;34(1):1–10. doi: 10.1017/wet.2019.73

[10] Reddy KN. Effects of Cereal and Legume Cover Crop Residues on Weeds, Yield, and Net Return in Soybean (Glycine max). Weed Technology. 2001;15(4):660–8.

[11] Thelen KD, Mutch DR, Martin TE. Utility of Interseeded Winter Cereal Rye in Organic Soybean Production Systems. Agronomy Journal. 2004;96(1):281–4. doi: 10.2134/agronj2004.2810

[12] SARE. 2013–2014 COVER CROP SURVEY REPORT. 2014.

[13] Creamer NG, Dabney SM. Killing cover crops mechanically: Review of recent literature and assessment of new research results. American Journal of Alternative Agriculture. 2002;17(1):32–40.

[14] Raper RL, Simionescu PA, Kornecki TS, Price AJ, Reeves DW. Reducing Vibration While Maintaining Efficacy of Rollers to Terminate Cover Crops. Applied Engineering in Agriculture. 2004;20(5):581–4. doi: 10.13031/2013.17458

[15] Kornecki TS, Price AJ, Raper RL, Arriaga FJ. New roller crimper concepts for mechanical termination of cover crops in conservation agriculture. Renewable Agriculture and Food Systems. 2009;24(3):165–73.

[16] Ashford DL, Reeves DW. Use of a mechanical roller-crimper as an alternative kill method for cover crops. American Journal of Alternative Agriculture. 2003;18(1):37–45. Epub 2009/10/30. doi: 10.1079/AJAA200232

[17] Wayman S, Kissing Kucek L, Mirsky SB, Ackroyd V, Cordeau Sp, Ryan MR. Organic and conventional farmers differ in their perspectives on cover crop use and breeding. Renewable Agriculture and Food Systems. 2017;32(4):376–85. doi: 10.1017/S1742170516000338

[18] Pittman KB, Flessner ML, Cahoon CW, Bamber KW. Herbicide Options to Terminate Winter Cover Crops.. In: Extension VC, editor. 2019.

[19] Oliveira MC, Butts L, Werle R. Assessment of Cover Crop Management Strategies in Nebraska, US. Agriculture. 2019;9(6). doi: 10.3390/agriculture9060124

[20] Palhano MG, Norsworthy JK, Barber T. Evaluation of Chemical Termination Options for Cover Crops. Weed Technology. 2018;32(3):227–35. doi: 10.1017/wet.2017.113

[21] Askew MC, Cahoon CW, Flessner ML, VanGessel MJ, Langston DB, Ferebee JH. Chemical termination of cover crop rapeseed. Weed Technology. 2019;33(5):686–92. doi: 10.1017/wet.2019.50

[22] Pittman KB, Cahoon CW, Bamber KW, Rector LS, Flessner ML. Herbicide selection to terminate grass, legume, and brassica cover crop species. Weed Technology. 2019;34(1):48–54. doi: 10.1017/wet.2019.107

[23] Reed HK, Karsten HD, Curran WS, Duiker SW, Tooker JF. Planting green effects on corn and soybean production. Agronomy Journal. 2019;111(5):2314–25. doi: 10.2134/agronj2018.11.0711

[24] Schramski JA, Sprague CL, Renner KA. Effects of fall-planted cereal cover-crop termination time on glyphosate-resistant horseweed (Conyza canadensis) suppression. Weed Technology. 2020;35(2):223–33. Epub 2020/09/14. doi: 10.1017/wet.2020.103

[25] Singh V, Rana A, Bishop M, Filippi AM, Cope D, Rajan N, et al. Chapter Three—Unmanned aircraft systems for precision weed detection and management: Prospects and challenges. In: Sparks DL, editor. Advances in Agronomy. 159: Academic Press; 2020. p. 93–134.

[26] Duddu HSN, Johnson EN, Willenborg CJ, Shirtliffe SJ. High-Throughput UAV Image-Based Method Is More Precise Than Manual Rating of Herbicide Tolerance. Plant Phenomics. 2019;2019:1–9. doi: 10.34133/2019/6036453 33313532 PMC7706330

[27] Taylor J, Wood G, Thomas G. Mapping yield potential with remote sensing. Precision Agriculture. 1997;1:713–20.

[28] Panda SS, Ames DP, Panigrahi S. Application of Vegetation Indices for Agricultural Crop Yield Prediction Using Neural Network Techniques. Remote Sensing. 2010;2(3):673–96. doi: 10.3390/rs2030673

[29] Jordan CF. Derivation of Leaf-Area Index from Quality of Light on the Forest Floor. Ecology. 1969;50(4):663–6.

[30] Prabhakara K, Hively WD, McCarty GW. Evaluating the relationship between biomass, percent groundcover and remote sensing indices across six winter cover crop fields in Maryland, United States. International Journal of Applied Earth Observation and Geoinformation. 2015;39:88–102. doi: 10.1016/j.jag.2015.03.002

[31] Jennewein JS, Lamb BT, Hively WD, Thieme A, Thapa R, Goldsmith A, et al. Integration of Satellite-Based Optical and Synthetic Aperture Radar Imagery to Estimate Winter Cover Crop Performance in Cereal Grasses. Remote Sensing [Internet]. 2022; 14(9).

[32] Hunt ER Jr, Daughtry CST, Mirsky SB, Hively WD. Remote sensing with simulated unmanned aircraft imagery for precision agriculture applications. IEEE Journal of Selected Topics in Applied Earth Observations and Remote Sensing. 2014;7(11). doi: 10.1109/JSTARS.2014.2317876

[33] Oseland E, Shannon K, Zhou J, Fritschi F, Bish MD, Bradley KW. Evaluating the Spectral Response and Yield of Soybean Following Exposure to Sublethal Rates of 2,4-D and Dicamba at Vegetative and Reproductive Growth Stages. Remote Sensing. 2021;13(18). doi: 10.3390/rs13183682

[34] Zhang J, Huang Y, Reddy KN, Wang B. Assessing crop damage from dicamba on non-dicamba-tolerant soybean by hyperspectral imaging through machine learning. Pest management science. 2019;75(12):3260–72. doi: 10.1002/ps.5448 31006969

[35] Huang Y, Thomson SJ, Ortiz BV, Reddy KN, Ding W, Zablotowicz RM, et al. Airborne remote sensing assessment of the damage to cotton caused by spray drift from aerially applied glyphosate through spray deposition measurements. Biosystems Engineering. 2010;107(3):212–20. 10.1016/j.biosystemseng.2010.08.003.

[36] Wang F-m Huang J-f, Tang Y-l Wang X-z. New Vegetation Index and Its Application in Estimating Leaf Area Index of Rice. Rice Science. 2007;14(3):195–203. doi: 10.1016/s1672-6308(07)60027-4

[37] Hancock DW, Dougherty CT. Relationships between Blue‐ and Red‐based Vegetation Indices and Leaf Area and Yield of Alfalfa. Crop Science. 2007;47(6):2547–56. doi: 10.2135/cropsci2007.01.0031

[38] Gitelson AA, Gritz † Y, Merzlyak MN. Relationships between leaf chlorophyll content and spectral reflectance and algorithms for non-destructive chlorophyll assessment in higher plant leaves. Journal of Plant Physiology. 2003;160(3):271–82. doi: 10.1078/0176-1617-00887 12749084

[39] Hunt ER, Daughtry CST, Eitel JUH, Long DS. Remote Sensing Leaf Chlorophyll Content Using a Visible Band Index. Agronomy Journal. 2011;103(4):1090–9. doi: 10.2134/agronj2010.0395

[40] Gitelson A, Merzlyak MN. Quantitative estimation of chlorophyll- a using reflectance spectra: Experiments with autumn chestnut and maple leaves. Journal of Photochemistry & Photobiology, B: Biology. 1994;22(3):247–52. doi: 10.1016/1011-1344(93)06963-4

[41] Sripada RP, Heiniger RW, White JG, Meijer AD. Aerial Color Infrared Photography for Determining Early In-Season Nitrogen Requirements in Corn. Agronomy Journal. 2006;98(4):968–77. doi: 10.2134/agronj2005.0200

[42] Tucker CJ. Red and photographic infrared linear combinations for monitoring vegetation. Remote Sensing of Environment. 1979;8(2):127–50. doi: 10.1016/0034-4257(79)90013-0

[43] Sperry BP, Lawrence BH, Golden BR, Edwards HM, Bond JA, Reynolds DB. Corn (Zea mays L.) response to sublethal rates of paraquat and fomesafen at vegetative growth stages. Weed Technology. 2019;33(4):595–600. doi: 10.1017/wet.2019.28

[44] Childers JT. Sensitivity of winter wheat (*Triticum aestivum* L.) to quizalofop-p-ethyl in central Oklahoma and Kansas.: Oklahoma State University; 2020.

[45] Raven AB, Todd AG, Franck ED. Low Temperature Delays Metabolism of Quizalofop in Resistant Winter Wheat and Three Annual Grass Weed Species2022; 3.

[46] Smeda RJ, Putnam AR. Influence of temperature, rainfall, grass species, and growth stage on efficacy of fluazifop. Weed Technology. 1990;4:349–55.

[47] Matzrafi M, Seiwert B, Reemtsma T, Rubin B, Peleg Z. Climate change increases the risk of herbicide-resistant weeds due to enhanced detoxification. Planta: An International Journal of Plant Biology. 2016;244(6):1217–27. doi: 10.1007/s00425-016-2577-4 27507240

[48] Kornecki TS, Arriaga FJ, Price AJ. Roller type and operating speed effects on rye termination rates, soil moisture, and yield of sweet Corn in a No-till system. HortScience. 2012;47(2):217–23.

[49] Kornecki TS. Influence of Recurrent Rolling/Crimping on Cover Crop Termination, Soil Strength and Yield in No-Till Cotton. AgriEngineering. 2020;2(4):631–48. doi: 10.3390/agriengineering2040042

[50] Mirsky SB, Curran WS, Mortensen DA, Ryan MR, Shumway DL. Control of Cereal Rye with a Roller/Crimper as Influenced by Cover Crop Phenology. Agronomy Journal. 2009;101(6):1589–96. doi: 10.2134/agronj2009.0130

[51] Pittman KB, Barney JN, Flessner ML. Cover crop residue components and their effect on summer annual weed suppression in corn and soybean. Weed Science. 2020;68(3):301–10. doi: 10.1017/wsc.2020.16

[52] Sievers T, Cook RL. Aboveground and Root Decomposition of Cereal Rye and Hairy Vetch Cover Crops. Soil Science Society of America Journal. 2018;82(1):147–55. doi: 10.2136/sssaj2017.05.0139

[53] Mischler R, Duiker SW, Curran WS, Wilson D. Hairy Vetch Management for No-Till Organic Corn Production. Agronomy Journal. 2010;102(1):355–62. doi: 10.2134/agronj2009.0183

[54] Price AJ, Duzy L, McElroy JS, Li S. Evaluation of Organic Spring Cover Crop Termination Practices to Enhance Rolling/Crimping. Agronomy. 2019;9(9). doi: 10.3390/agronomy9090519

[55] Shen M, Chen J, Zhu X, Tang Y. Yellow flowers can decrease NDVI and EVI values: Evidence from a field experiment in an alpine meadow. Canadian journal of remote sensing. 2009;35:99–106. doi: 10.5589/m09-003

[56] Dixon DJ, Callow JN, Duncan JMA, Setterfield SA, Pauli N. Satellite prediction of forest flowering phenology. Remote Sensing of Environment. 2021;255:112197. 10.1016/j.rse.2020.112197.

[57] Aklilu Tesfaye A, Gessesse Awoke B. Evaluation of the saturation property of vegetation indices derived from sentinel-2 in mixed crop-forest ecosystem. Spatial Information Research. 2021;29(1):109–21. doi: 10.1007/s41324-020-00339-5

